# High-threshold Ca^2+^ channels behind gamma band activity in the pedunculopontine nucleus (PPN)

**DOI:** 10.14814/phy2.12431

**Published:** 2015-06-24

**Authors:** Brennon Luster, Stasia D'Onofrio, Francisco Urbano, Edgar Garcia-Rill

**Affiliations:** 1 Center for Translational Neuroscience, University of Arkansas for Medical SciencesLittle Rock, Arkansas; 2 IFIBYNE-CONICET, University of Buenos AiresBuenos Aires, Argentina

**Keywords:** Arousal, gamma oscillations, N-type, P/Q-type, rats, waking

## Abstract

The pedunculopontine nucleus (PPN) is part of the Reticular Activating System, and active during waking and REM sleep. Previous results showed that all PPN cells plateau at gamma frequencies and intrinsic membrane oscillations in PPN neurons are mediated by high-threshold N- and P/Q-type Ca^2+^ channels. The present study was designed to determine whether some PPN cells have only N-, only P/Q-, or both N- and P/Q-type Ca^2+^ channels. We used patch-clamp recordings in PPN cells in slices from anesthetized rat pups in the presence of synaptic receptor blockers (SB) and Tetrodotoxin (TTX), and applied ramps to induce intrinsic membrane oscillations. We found that all PPN cell types showed gamma oscillations in the presence of SB+TTX when using current ramps. In 50% of cells, the N-type Ca^2+^ channel blocker *ω*-Conotoxin-GVIA (*ω*-CgTx) reduced gamma oscillation amplitude, while subsequent addition of the P/Q-type blocker *ω*-Agatoxin-IVA (*ω*-Aga) blocked the remaining oscillations. Another 20% manifested gamma oscillations that were not significantly affected by the addition of *ω*-CgTx, however, *ω*-Aga blocked the remaining oscillations. In 30% of cells, *ω*-Aga had no effect on gamma oscillations, while *ω*-CgTx blocked them. These novel results confirm the segregation of populations of PPN cells as a function of the calcium channels expressed, that is, the presence of cells in the PPN that manifest gamma band oscillations through only N-type, only P/Q-type, and both N-type and P/Q-type Ca^2+^ channels.

## Introduction

The pedunculopontine nucleus (PPN) is a component of the brainstem Reticular Activating System (RAS), and is the only RAS nucleus active during both waking and paradoxical or rapid eye movement (REM) sleep. The EEG shows low-amplitude, high-frequency activity in the beta (20–30 Hz) and gamma (30–90 Hz) frequencies, and it appears to be similar during waking and REM sleep, despite the fact that these are markedly different states. Gamma frequency oscillations during waking are thought to participate in sensory perception, problem solving, and memory (Eckhorn et al. 1988; Gray and Singer 1989; Jones 2007; Boss et al. 2009; Phillips and Takeda 2009; Palva et al. 2010). Gamma frequency oscillations are also evident during REM sleep, when our skeletal muscles undergo atonia and we experience neurogenic surrealistic dreaming (Llinás and Paré 1991).

We previously reported that, regardless of transmitter type, whether cholinergic, glutamatergic, or GABAergic, all PPN neurons fired at beta/gamma band frequency, but no higher, when subjected to depolarizing steps (Simon et al. 2010). These oscillations were mediated by high-threshold, voltage-dependent, N- and P/Q-type Ca^2+^ channels; both voltage-dependent high-threshold N- and P/Q-type channels mediate the depolarizing phase of gamma band oscillations in the PPN (Kezunovic et al. 2011). However, these findings did not indicate whether some PPN cells manifest this high-frequency activity via both channel types, only P/Q-type channels or only N-type channels.

The present study was designed to determine whether there is a segregation of populations of PPN cells as a function of the Ca^2+^ channels expressed, that is, if some PPN cells have only N-, only P/Q-, or both N- and P/Q-type channels, and determine if any have neither one of these channels. We also assessed the effects of these channels on Ca^2+^ currents in PPN cells. A thorough determination of PPN cell types is essential to understand the differential role of cells manifesting different high-threshold Ca^2+^ channels mediating high-frequency activity in the PPN. Once we know which types of cells with Ca^2+^ channels reside in the PPN, we can identify the intracellular mechanisms mediating high-frequency activity, with the aim of differentially manipulating cells with different Ca^2+^ channel types to promote or inhibit one type of activity or state versus the other, that is, to promote or reduce waking versus REM sleep.

## Methods

### Slice preparation

Rodent pups aged 11–17 days from adult timed-pregnant Sprague–Dawley rats (280–350 g) were anesthetized with ketamine (70 mg/kg, i.m.) until the tail pinch reflex was absent. This age range was selected due to the developmental decrease in REM sleep of the rat that occurs between 10 and 30 days (Jouvet-Mounier et al. 1970). This enabled sampling from a baseline period (9–13 days), before the epoch of the greatest transitions that peak at 14–16 days and continue until >20 days, as determined by our previous work on the PPN (Garcia-Rill et al. 2008). Pups were decapitated and the brain was rapidly removed and cooled in oxygenated sucrose-artificial cerebrospinal fluid (sucrose-aCSF). The sucrose-aCSF consisted of (in mmol/L): 233.7 sucrose, 26 NaHCO_3_, 3 KCl, 8 MgCl_2_, 0.5 CaCl_2_, 20 glucose, 0.4 ascorbic acid, and 2 sodium pyruvate. Sagittal sections (400 *μ*m) containing the PPN were cut and slices were allowed to equilibrate in normal aCSF at room temperature for 1 h. The aCSF was composed of (in mmol/L): 117 NaCl, 4.7 KCl, 1.2 MgCl_2_, 2.5 CaCl_2_, 1.2 NaH_2_PO_4_, 24.9 NaHCO_3_, and11.5 glucose. Slices were recorded at 37°C while perfused (1.5 mL/min) with oxygenated (95% O_2_- 5% CO_2_) aCSF in an immersion chamber for patch-clamp studies as previously described (Kezunovic et al. 2011, 2012, 2013). The superfusate contained the following synaptic receptor antagonists: the selective NMDA receptor antagonist 2-amino-5-phosphonovaleric acid (APV, 40 *μ*mol/L), the competitive AMPA/Kainate glutamate receptor antagonist 6-cyano-7-nitroquinoxaline-2,3-dione (CNQX, 10 *μ*mol/L), the glycine receptor antagonist strychnine (STR, 10 *μ*mol/L), and the specific GABA_A_ receptor antagonist gabazine (GBZ, 10 *μ*mol/L), collectively referred to here as synaptic blockers (SB). All experimental protocols were approved by the Institutional Animal Care and Use Committee of the University of Arkansas for Medical Sciences and were in agreement with the National Institutes of Health guidelines for the care and use of laboratory animals.

### Whole-cell patch-clamp recordings

Differential interference contrast optics was used to visualize neurons using an upright microscope (Nikon FN-1; Nikon Instruments, Melville, NY). Whole-cell recordings were performed using borosilicate glass capillaries pulled on a P-1000 puller (Sutter Instrument Company, Novato, CA) and filled with a high-K^+^ intracellular solution, designed to mimic the intracellular electrolyte concentration, of (in mmol/L): 124 K-gluconate, 10 HEPES, 10 phosphocreatine di tris, 0.2 EGTA, 4 Mg_2_ATP, 0.3 Na_2_GTP; or a high-Cs^+^ (125 mmol/L)/QX-314 (5 mmol/L) intracellular solution (in mmol/L): 120 CsMeSO_3_, 40 HEPES, 1 EGTA, 10 TEA-Cl, 4 Mg-ATP, 0.4 mmol/L GTP, 10 Phosphocreatine, 2 MgCl_2_. Osmolarity was adjusted to ∽270–290 mOsm and pH to 7.3. The pipette resistance was 2–5 MΩ. All recordings were made using a Multiclamp 700B amplifier (Molecular Devices, Sunnyvale, CA) in both current and voltage-clamp mode. Digital signals were low pass filtered at 2 kHz, and digitized at 5 kHz using a Digidata-1440A interface and pClamp10 software (Molecular Devices). The recording region was located mainly in the *pars compacta* in the posterior PPN, immediately dorsal to the superior cerebellar peduncle. This area of PPN has been shown to have the highest density of cells (Wang and Morales 2009; Ye et al. 2010). In addition, our previous studies determined that all PPN neurons manifest beta/gamma band oscillations regardless of neurotransmitter type, and that only cells within the PPN manifest such oscillations, making localization standard without the need for immunocytochemical labeling (Simon et al. 2010; Kezunovic et al. 2011, 2013; Hyde et al. 2013). Gigaseal and further access to the intracellular neuronal compartment was achieved in a voltage-clamp configuration mode, setting the holding potential at −50 mV (i.e., near the average resting membrane potential of PPN neurons). Within a short time after rupturing the membrane, the intracellular solution reached equilibrium with the pipette solution without significant changes in either series resistance (ranging 4–14 MΩ) or membrane capacitance values. The configuration was then changed to current clamp mode and PPN cell type I (low threshold spike-LTS current), type II PPN cells (I_a_ current), and type III PPN neurons (LTS + I_a_ currents) were identified as previously described (Kezunovic et al. 2011, 2013; Hyde et al. 2013). Average bridge values in current clamp were 12 ± 1 MΩ (*n* = 30). The membrane potential was then depolarized using a 1-sec long depolarizing ramp.

### Ca^2+^ currents

Voltage-dependent Ca^2+^ currents were studied using a high-Cs^+^/QX314 pipette solution (in mmol/L: CsMeSO_3_, 120; HEPES, 40; EGTA, 1; tetraethyl ammonium chloride (TEA-Cl), 10; Mg-ATP, 4; mm GTP, 0.4; phosphocreatine, 10; and MgCl_2_, 2. Cesium and TEA-Cl are widely used K^+^ channel blockers. Ca^2+^ currents were recorded in the presence of extracellular SBs listed above, and the Na^+^ and K^+^ channel blockers tetrodotoxin (TTX, 3 *μ*mol/L) and TEA-Cl (25 mmol/L), respectively, as previously described (Kezunovic et al. 2011, 2012, 2013). Since PPN neurons have long dendritic arborizations that can be difficult to depolarize, we combined QX-314 in the pipette (to block Na^+^ currents from the inside) and superfused TTX (to block Na^+^ currents from the outside) in order to prevent voltage-gated Na^+^ currents from being elicited during depolarization. We used two different protocols to demonstrate the effects on Ca^2+^ currents. The first used a square voltage step to generate PPN neuronal peak Ca^2+^ currents from a holding potential of −50 mV, and applied a depolarization step to 0 mV. This allowed rapid assessment of only high-threshold currents, as in Fig.3A–C. Using a −50 mV holding potential allowed us to inactivate T-type Ca^2+^ channels, centering these recordings on the high-threshold Ca^2+^ currents (Kezunovic et al. 2011, 2013; Hyde et al. 2013). The other protocol used a hyperpolarizing step to −70 mV from the holding potential of −50 mV, then depolarized in 10 mV steps up to 0 mV. This allowed activation of low threshold Ca^2+^ currents, and the calculation of a full current-voltage plot. Both series resistance and liquid junction potential were compensated (>14 kHz correction bandwidth; equivalent to <10 msec lag). No significant rundown due to intracellular dialysis of PPN neuron supra- or subthreshold activity was observed during our recording period for control PPN neurons (up to 30 min). Fast compensation was used to maintain the series resistance <9 MΩ.

### Drug application

Bath-applied drugs such as SBs were administered to the slice via a peristaltic pump (Cole-Parmer, Vernon Hills, IL), and a three-way valve system such that solutions reached the slice 1.5 min after the start of application. TTX, TEA-Cl, Cs+, cadmium, and the synaptic receptor blockers listed above, were purchased from Sigma Aldrich (St. Louis, MO). Channel blockers were purchased from Alomone laboratories (Alomone.com). We used *ω*-Agatoxin-IVA (*ω*-AgA; 100 nmol/L), a specific P/Q-type channel blocker, and *ω*-Conotoxin-GVIA (*ω*-CgTx; 2.5 *μ*mol/L), a specific N-type channel blocker. To elicit oscillations, the membrane potential was depolarized using a 1-sec duration ramp current clamp protocol. The membrane resting potential was kept at −50 mV to inactivate T-type calcium channels, and the amount of current injected during 1 sec ramps was adjusted to depolarize PPN membrane throughout the voltage range in which high-threshold channels start opening and reach their peak amplitude (from −30 to 0 mV; Kezunovic et al. 2011). We previously showed that the peak effect of *ω*-CgTx on oscillations was reached after 10 min of superfusion, while the effects of *ω*-AgA took ∽15 min to peak (Kezunovic et al. 2011). Using Ca^2+^ imaging, after 10 min exposures to *ω*-CgTx and *ω*-AgA superfusion, no change in intracellular Ca^2+^ concentration was observed after a 1-sec duration ramp in current clamp (Hyde et al. 2013). Since P/Q-type channels are key elements underlying gamma oscillation in PPN neurons (Kezunovic et al. 2011; Hyde et al. 2013), we initially used *ω*-CgTx superfusion (to block N-type channels first) and followed by *ω*-AgA. The total superfusion time was 20–25 min. If all of the oscillations were blocked by initial application of *ω*-CgTx, the cell was assumed to have only N-type channels. If the oscillations were reduced or a frequency shift due to increased input resistance occurred, then the oscillations were assumed to be due to both N- and P/Q-type channels. The latter was confirmed if the addition of *ω*-AgA blocked the remaining oscillations. However, if after *ω*-CgTx the oscillations remained the same, they were assumed to be due only to P/Q-type Ca^2+^ channels, and were confirmed with the addition of *ω*-Aga to block the oscillations. We also recorded from 10 cells using each blocker only to determine if there was an order effect or a differential effect of the toxins. As described below, the results of using only one blocker were consistent with those using two blockers, that is, there was no order effect in determining which channel mediated beta/gamma oscillations.

During Ca^2+^ current (I_Ca_) recordings, a small component (<1%) of total currents remained unaffected after the combination of *ω*-CgTx and *ω*-AgA toxins was used. This component was blocked using cadmium (Cad), and has been previously described to be related to Ca^2+^ channels located at very distal PPN dendrites that can be difficult to reach (Hyde et al. 2013). The existence of such a small component of I_Ca_ was due to experimental conditions (i.e., after K^+^ and Na^+^ channels were blocked using high-Cs^+^ intracellular and TEA-Cl containing extracellular solutions (see Kezunovic et al. 2011; Hyde et al. 2013), these conditions can amplify very small I_Ca_ at less accessible PPN dendrites for the toxins to reach. However, *ω*-CgTx and *ω*-AgA toxins were able to maximally block gamma oscillations using normal current clamp physiological conditions.

### Data analysis

Off-line analyses were performed using Clampfit software (Molecular Devices, Sunnyvale, CA). As stated above, we used 1-sec duration ramps applied every 10 min in current clamp in the presence of SBs and TTX to record membrane oscillations. Peak oscillatory amplitude was analyzed by first filtering each ramp recording and measuring the three highest amplitude oscillations to derive a mean amplitude induced during each ramp. The mean peak frequency of the same three oscillations was filtered (low pass 10 Hz, high pass 120 Hz), and measured to derive a mean frequency during the three highest amplitude oscillations in each ramp. The power of each frequency was also analyzed by composing a power spectrum for the frequencies in the entire ramp, giving a measure of peak power for frequency. Comparisons between groups were carried out using one-way or two-way ANOVA, with Bonferroni post hoc testing for multiple comparisons.

## Results

Whole-cell patch-clamp recordings were performed on 75 PPN neurons. We tested 50 PPN neurons using 1-sec long depolarizing current ramps, in the presence of SBs and TTX. Thirty of these were tested using *ω*-CgTx followed by *ω*-Aga, another 10 cells were tested only using *ω*-CgTx, and 10 more using only *ω*-Aga. We then tested 25 neurons to study Ca^2+^ currents: 20 were tested using a single-step protocol to determine the differential effects of channel blockers, while five were tested using a multiple ramp protocol to determine the effects of each Ca^2+^ channel blocker across multiple depolarizing levels and the effects of the unspecific Ca^2+^ channel blocker Cad. PPN neurons showed gamma oscillations when voltage clamped at holding potentials above −30 mV, suggesting that their origin may be spatially located beyond voltage-clamp control. No difference in average resting membrane potential was observed among PPN neuronal types I, II, or III (Simon et al. 2010; Kezunovic et al. 2011, 2013; Hyde et al. 2013). All PPN cells responded to one or both channel blockers, suggesting that the PPN does not contain cells that do not have high-threshold Ca^2+^ channels.

### Cells with both N- and P/Q-type channels

**Figure 1:**
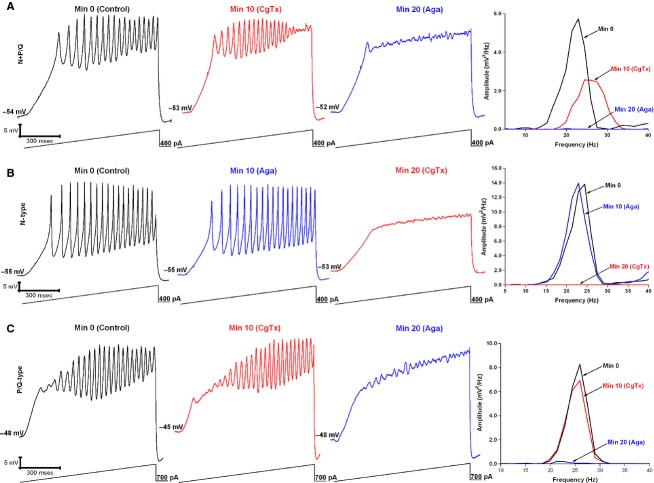
Cells in the PPN mediate gamma band activity through N- and P/Q-, N only, and P/Q only type channels. (A) Membrane oscillations recorded in a PPN cell during 1-sec long ramps in the presence of SB + TTX (left record, black). Following superfusion with *ω*-CgTx for 10 min, oscillation amplitude was reduced (middle record, red). Thereafter, *ω*-Aga was superfused for 10 min blocking the remaining oscillations (right record, blue). At right is a power spectrum of the records from the neuron shown in A displaying amplitude and frequency of ramp-induced oscillations before (black record, beta/gamma range), after *ω*-CgTx (red record, reduced oscillations), and following *ω*-Aga (blue record, blocked remaining oscillations). (B) Membrane oscillations recorded in a different PPN cell during 1 sec long ramps in the presence of SB+TTX (left record, black). *ω*-Aga applied into the bath for 10 min caused no significant effect on the membrane oscillations (middle record, blue). *ω*-CgTx was then superfused for 10 min, causing a complete blockade of the membrane oscillations (right record, red). At right is a power spectrum of the record from this cell shown in B displaying amplitude and frequency of ramp-induced oscillations before application of toxins (black record, beta/gamma range), after *ω*-Aga (blue record, no effect), and *ω*-CgTx (red record, blocked oscillations). (C) Membrane oscillations recorded in a third PPN cell during 1 sec long ramps in the presence of SB + TTX (left record, black). *ω*-CgTx was applied for 10 min causing no significant effect on the oscillations (middle record, red). *ω*-Aga then was superfused for 10 min causing a complete blockade of the membrane oscillations (right record, blue). At right is a power spectrum of the record of the cell shown in C displaying amplitude and frequency of ramp-induced oscillations before application of toxins (black record, beta/gamma range), after *ω*-CgTx (red record, no effect), and *ω*-Aga (blue record, blocked oscillations).

Thirty cells were recorded from the PPN to test the hypothesis that there are cells in the PPN that can manifest gamma band oscillations through N- and/or P/Q-type channels, as determined by superfusion with *ω*-CgTx followed by *ω*-Aga (Fig.1). Figure1A shows the response of a PPN cell to a 1-sec ramp to induce membrane oscillations in the presence of SB and TTX (black record). After 10 min exposure to *ω*-CgTx, the oscillations were reduced but not eliminated (red record). The remaining oscillations were blocked by 20 min exposure to *ω*-Aga (blue record). This neuron was assumed to have both N- and P/Q-type channels.

Figure1B shows the responses of another cell that manifested ramp-induced oscillations (black record), which were not affected by the addition of *ω*-Aga (blue record), but were completely blocked by the addition of *ω*-CgTx (red record). This neuron was assumed to have only N-type channels.

Figure1C shows the responses of another PPN cell manifesting ramp-induced oscillations (black record) that were unaffected by addition of *ω*-CgTx (red record), but were completely abolished by *ω*-Aga (blue record). This PPN neuron was assumed to have only P/Q-type channels.

Fifteen cells (50%) had both N- and P/Q-type channels (Table1), and could be any of the three PPN cell types (Type 1 LTS, Type II I_a_ current, Type III LTS + I_a_). Their mean oscillation amplitude was 2.3 ± 0.3 mV with a mean oscillation frequency of 37 ± 3 Hz at min 0. After 10 min of *ω*-CgTx, the mean oscillation amplitude was reduced by 40% to 1.4 ± 0.6 mV (one-way ANOVA df = 14, *t* = −2.17, *P* < 0.05). After 20 min exposure to *ω*-Aga, the oscillation amplitude was reduced by an additional 60% to 0.9 ± 0.1 mV (Fig.1A; one-way ANOVA df = 14, *t* = −3.46, *P* < 0.05). Oscillation frequency in the control condition was 37 ± 3 Hz, but remained unaffected (<10% change) after *ω*-CgTx (39 ± 4 Hz) (df = 29, *t* = 0.49, ns), and after *ω*-Aga (42 ± 5 Hz) (df = 29, *t* = 0.82, ns).

**Table 1:** Responses of PPN cells by cell type, and oscillation amplitude and frequency, following exposure to the specific N-type channel blocker *ω*-CgTx, or the specific P/Q-type channel blocker *ω*-Aga

Channel type	Cell type	Osc. Amp. (mV)	Osc. Amp. (mV) +CgTx	Osc. Amp. (mV) +Aga	Osc. Freq. (Hz)	Osc. Freq. (Hz) +CgTx	Osc. Freq. (Hz) +Aga
N+PQ	I - 1/15	2.25 ± 0.3	1.36 ± 0.61	0.90 ± 0.12	37 ± 3	39 ± 4	42 ± 5
15/30	II - 9/15		*↓40%*	↓60%		–	–
50%	III - 5/15		*P* < 0.05	*P* < 0.05			
N only	I - 1/9	2.08 ± 0.23	1.11 ± 0.16	1.44 ± 0.17	53 ± 9	50 ± 7	46 ± 8
9/30	II - 4/9		53%	NR		↓6%	↓14%
30%	III - 4/9		*P* < 0.05				
P/Q only	I - 1/6	1.29 ± 0.1	1.85 ± 0.26	0.89 ± 0.06	44 ± 8	32 ± 5	44 ± 8
6/30	II - 3/6		NR	↓31%		↓27%	–
20%	III - 2/6			*P* < 0.05			

### Cells with only N-type channels

Nine cells (30%) had only N-type channels (Table1), and these could be of any of the three PPN cell types (Type 1 LTS, Type II I_a_ current, Type III LTS + I_a_). Their mean oscillation amplitude was 2.1 ± 0.2 mV at min 0 (one-way ANOVA df = 17, *t* = −3.46, *P* < 0.05). These cells were not further affected by exposure to *ω*-Aga (1.4 ± 0.2 mV) (Fig.1B). These cells had a higher initial mean oscillation frequency than cells with other channel types (53 ± 9 Hz). *ω*-CgTx induced a small, nonsignificant decrease in mean oscillation frequency in these cells (50 ± 7 Hz) (df = 17, *t* = −0.21, ns), oscillation frequency was further decreased by *ω*-Aga (46 ± 8 Hz), but this decrease also was not statistically significant (df = 17, *t* = −0.62, ns).

### Cells with only P/Q-type channels

Six cells (20%) had only P/Q-type channels (Table1; Fig.1C), and could be of any of the three PPN cell types (Type 1 LTS, Type II I_a_ current, Type III LTS + I_a_). The mean oscillation amplitude was 1.3 ± 0.1 mV at min 0. After 10 min of exposure to *ω*-CgTx, these cells showed no significant reduction in their oscillation amplitude (1.9 ± 0.3 mV). However, after 10 min of *ω*-Aga, the mean amplitude was reduced by 31% to 0.9 ± 0.1 mV (one-way ANOVA df = 11, *t* = −2.67, *P* < 0.05). These cells had an initial mean oscillation frequency of 44 ± 8 Hz. Although *ω*-CgTx had no significant affect on mean oscillation ampliude, a nonsignificant reduction was seen in mean oscillation frequency 32 ± 9 Hz (df = 11, *t* = −1.35, ns). Mean oscillation frequency was unaffected by *ω*-Aga (44 ± 8 Hz) (df = 11, *t* = −0, ns).

**Figure 2:**
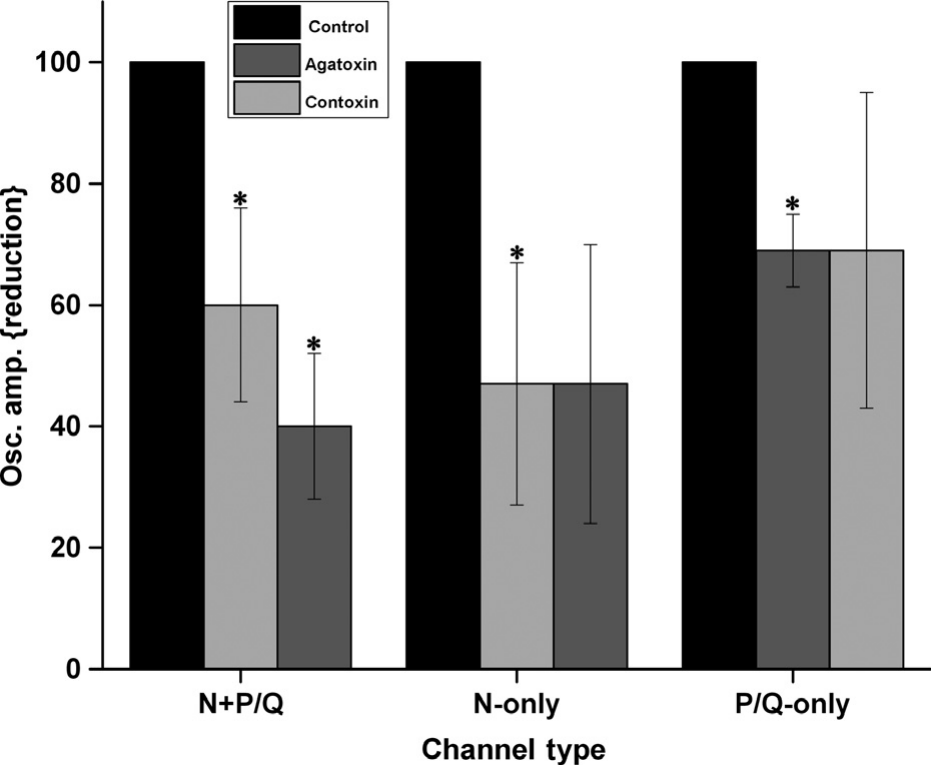
Reduction by toxins on the mean peak oscillation amplitude of each channel type. The bar graph shows the mean and standard error of the peak oscillation amplitude of cells within each channel type (left, N+P/Q *n* = 15; middle, N only *n* = 9; right, P/Q only *n* = 6) calculated by measuring the three highest amplitude oscillations after filtering to derive mean amplitude. Light Gray bars indicate the percentage of reduction caused by the addition of *ω*-CgTx. Dark gray bars indicate the percentage of reduction caused by the addition of *ω*-Aga. On the left, N+P/Q cells, there was a significant reduction in amplitude after *ω*-Aga, and an additional significant reduction after *ω*-CgTx. In the middle, N only cells showed a significant reduction after *ω*-CgTx, but no further reduction after *ω*-Aga. On the right, P/Q only cells showed a significant reduction after *ω*-Aga, but no further reduction after *ω*-CgTx, *P < 0.05.

Figure2 summarizes the effects on mean oscillation amplitude on all cells tested with both *ω*-Aga and *ω*-CgTx. Oscillation amplitude in control recordings was normalized to 100%, and the relative effect of each channel blocker plotted. In cells with both N- and P/Q-type channels, *ω*-Aga reduced oscillation amplitude by 40% (to 60% of control values), while *ω*-CgTx reduced amplitude by 60%. In cells with only N-type channels, *ω*-CgTx reduced amplitude by 50%, while *ω*-Aga had no effect in addition to the effect of *ω*-CgTx. In cells with only P/Q-type channels, *ω*-Aga reduced oscillation amplitude by 30% (to 70% of control values), while *ω*-CgTx had no further effect.

### Comparisons across cell types

When we compared the control oscillation amplitude between N+P/Q (2.3 ± 0.3 mV), N only (2.1 ± 0.2 mV), and P/Q only (1.3 ± 0.1 mV) cell types, there were no significant differences (df = 2, *F* = 5.35, *P* > 0.05, ns), although the amplitude of the oscillations in P/Q only type cells was (nonsignificantly) lower. When we compared the pretreatment, control oscillation frequency between N+P/Q (37 ± 3 Hz), N only (53 ± 9 Hz), and P/Q only (44 ± 8 Hz), there were significant differences (df = 2, *F* = 5.25, *P* < 0.03), with the frequency of N+P/Q cells being lower than those of N only and P/Q only cells.

#### Cells tested only with *ω*-Aga (*n* = 10) and *ω*-CgTx (*n* = 10)

This protocol was employed to determine the effects of applying one versus the other toxin only. Both *ω*-CgTx and *ω*-Aga reduced the mean oscillation amplitude. However, *ω*-CgTx appeared to slightly reduce the mean oscillation frequency while *ω*-Aga appeared to slightly increase oscillation frequency. We discarded four cells that did not initially respond to *ω*-CgTx (that were probably P/Q only cells), and two that did not initially respond to *ω*-Aga (that were probably N only cells).

The effects of *ω*-CgTx were studied in 10 cells. The initial oscillation amplitude of 4.0 ± 0.9 mV was reduced by 41% to 2.4 ± 0.6 mV (not significantly different). At min 20, oscillation amplitude showed a further reduction by decreasing an additional 19% to 1.6 ± 0.2 mV (one-way ANOVA df = 19, *t* = 2.49, *P* < 0.05). These cells had an initial mean oscillation frequency of 46 ± 2 Hz. *ω*-CgTx induced a 15% decrease in frequency to 39 ± 4 Hz (nonsignificant difference).

The effects of *ω*-Aga only were also determined. The initial oscillation amplitude of 4.5 ± 1.4 mV was reduced by 60% to 1.8 ± 0.4 mV, but this reduction was not statistically significant. At min 20, oscillation amplitude showed a further reduction to 1.4 ± 0.3 mV (one-way ANOVA df = 17, *t* = −2.25, *P* < 0.05). These cells (*n* = 10) had an initial mean frequency of 36 ± 6 Hz. After 10 min exposure to *ω*-Aga, the mean frequency increased to 42 ± 4 Hz. At min 20, the mean frequency was reduced to 40 ± 4 Hz, but still remained near the mean oscillation frequency at min 10 (nonsignificant difference).

### Ca^2+^ currents

**Figure 3:**
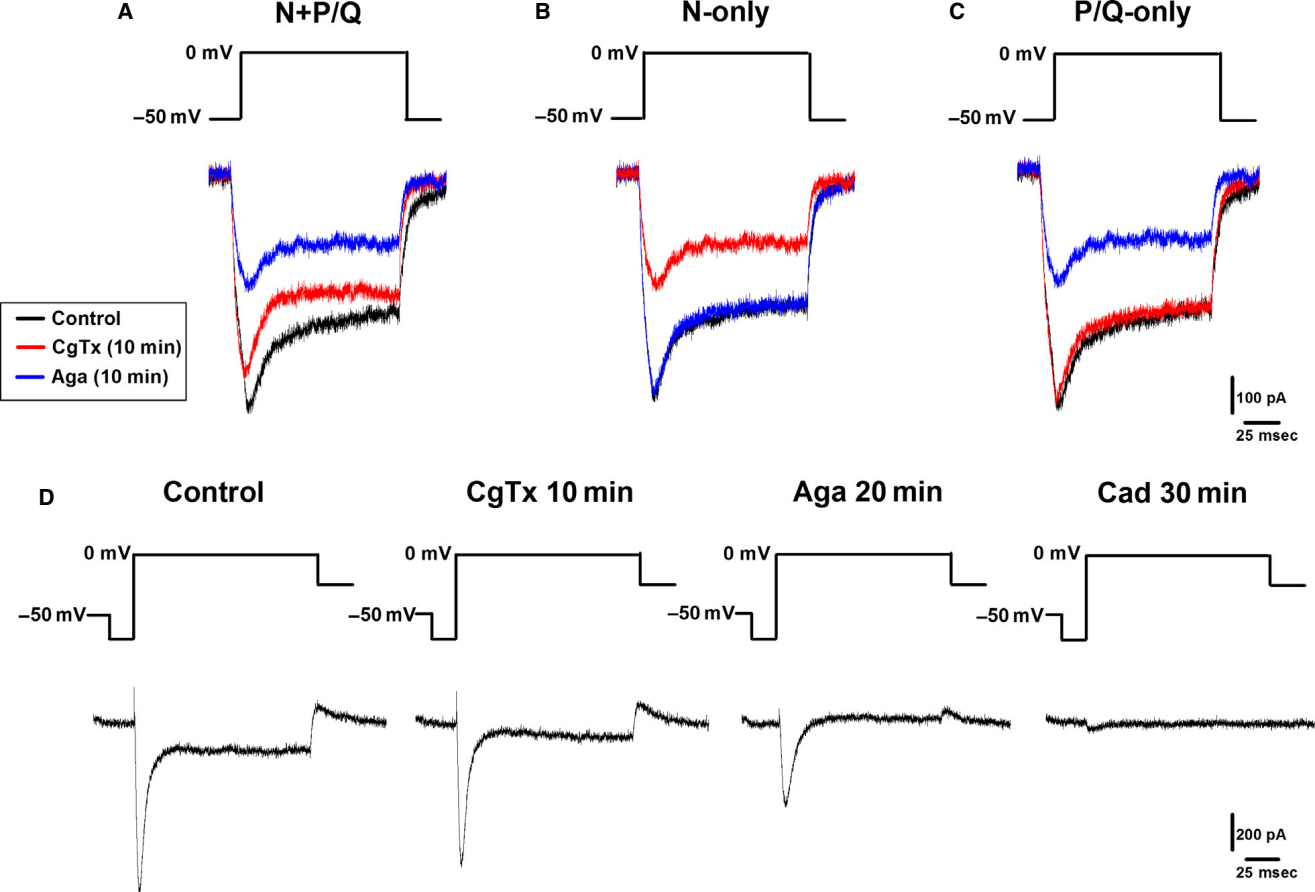
Ca^2+^ current recordings showing cells with N- and P/Q, only N-type, and only P/Q-type channels. (A) Using a depolarizing step at −50 mV, Ca^2+^ currents were recorded in the presence of SB+TTX+TEA-Cl from a cell with both N and P/Q- type channels (left panel) before (black record), after *ω*-CgTx (blue record, reduction) and *ω*-CgTx (red record, reduction). (B) Ca^2+^ currents recorded from a cell with only N-type channels (middle panel) before (black record), after *ω*-Aga (blue record, no effect) and *ω*-CgTx (red record, reduction). (C) Ca^2+^ currents were recorded from a cell with only P/Q- type channels (right panel) before (black record), after *ω*-CgTx (red record, no effect), and *ω*-Aga (red record, reduction). (D) Representative currents obtained using a depolarization protocol with multiple steps (see Methods), showing only the 0 mV step. Initial currents were recorded in the presence SB+TTX+TEA-Cl (left record). *ω*-Aga was superfused in the aCSF bath for 10 min causing a reduction in the current (second record). *ω*-CgTx (third record) was then superfused for 10 min causing a further reduction in the current, followed by the addition of Cadmium (right record) for 10 min leading to a complete block of the Ca^2+^ currents.

To evaluate the effects of *ω*-Aga and *ω*-CgTx on Ca^2+^ currents in PPN neurons (*n* = 25), voltage-clamp recordings were obtained using a square voltage step in combination with high Cesium/QX314 intracellular pipette solution, and superfused TTX, TEA-Cl, and SBs. The holding potential was initially clamped at −50 mV and then depolarized to 0 mV. This square voltage step was applied shortly after breaking into the cell and applied every 30 sec for 30 min. Figure3A, left side, shows the protocol used as well as Ca^2+^ currents of a PPN cell with both N- and P/Q-type channels. Control currents (black record) were obtained after applying the depolarizing step. *ω*-Aga (blue record) was added to the bath solution for 10 min, causing a reduction in the current. After 10 min of exposure *ω*-CgTx (red record), a further reduction in the current was seen, confirming the presence of both channel types in this cell. Figure3B, middle panel, shows the Ca^2+^ currents recorded in another cell with only N-type channels. Control recordings (black record) were obtained after applying a depolarizing step to 0 mV. After 10 min of *ω*-Aga (blue record) there was no significant effect on the current. However, after *ω*-CgTx (red record) was added for 10 min, there was a clear reduction in the current, suggesting this cell had only N-type channels. Conversely, Fig.3C, right side, shows the Ca^2+^ currents of a third cell determined to have only P/Q-type channels. Control currents (black record) were obtained after a depolarizing step. *ω*-CgTx (red record) was added to the bath solution for 10 min, however, no significant changes were observed in the current. The cell was then exposed to *ω*-Aga (blue record) for 10 min causing a reduction in the current, suggesting that this cell had only P/Q-type channels.

In the last group of cells tested (*n* = 5), Ca^2+^ currents were recorded using voltage-clamp depolarizing steps from −50 mV, then hyperpolarized to −70 mV, then depolarized to 0 mV in 10 mV steps (Fig.3B). Currents were recorded every 5 min for 30 min. Figure3D shows the depolarizing step protocol as well as Ca^2+^ currents from a cell with both N- and P/Q-type channels (left record). After 10 min exposure to *ω*-Aga (second record), the Ca^2+^ current showed an obvious reduction. The reduction was increased after 10 min exposure to *ω*-CgTx (third record). These results suggested that this cell had both N- and P/Q-type channels. Subsequent addition of Cad completely blocked the remaining tail current (right record), demonstrating that the Ca^2+^ tail currents were mediated by high-threshold Ca^2+^ channels.

**Figure 4:**
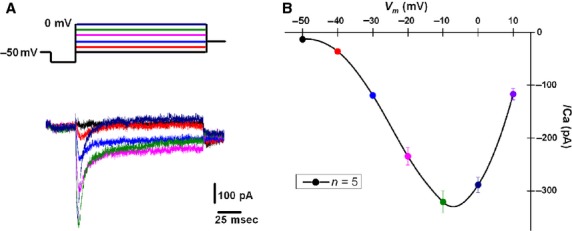
Average current-voltage (I–V) curve from PPN neurons. (A) Representative currents obtained in the presence of SB+TTX+TEA-Cl using a multiple step depolarization protocol (see Methods). (B) Average current-voltage (I–V) curve showing both low voltage- (T-type) and high voltage-activated currents in PPN neurons (*n* = 5). Note that high-threshold currents peaked at ∽−10 mV.

Figure4 shows the current-voltage plot of Ca^2+^ currents for the 5 PPN neurons in the last group of cells. The peak of the high-threshold Ca^2+^ currents was between −10 mV and 0 mV, as previously described (Kezunovic et al. 2011).

## Discussion

We have previously shown that every cell in the PPN fires maximally at gamma band frequencies but no higher (Simon et al. 2010). Moreover, it was shown that such high-frequency activity was mediated by high-threshold voltage-dependent P/Q-, and also N-, type Ca^2+^ channels (Kezunovic et al. 2011). However, these results did not indicate whether there is a segregation of cells as a function of the channels expressed, that is, if some PPN cells could manifest this high frequency activity through only P/Q-type channels and/or only N-type channels. Our findings confirm the presence of cells in the PPN that mediate gamma band oscillations through only N-type channels, only P/Q-type channels, or both. Most cells had both N- and P/Q-type channels, and no cell was found to have neither channel. In general, cells tested with both N- and P/Q-type channel blockers underwent partial reductions in oscillation *amplitude* that were additive (Figs.1 and 2), that is, each channel blocker reduced oscillation amplitude. Other cells manifested only N-type or P/Q-type channels. These results emphasize the segregation of three groups of PPN cells according to channel type.

### Functional Implications

Recordings of PPN neurons in vivo have reported three types of cell activity in the cat and rat, “REM on”, “Wake/REM on”, and “Wake on” (Sakai et al. 1990; Steriade et al. 1990; Datta and Hobson 1994; Datta and Siwek 2002; Boucetta et al. 2014). Some studies in vivo identified multiple types of thalamic-projecting PPN cells distinguished by their firing properties relative to ponto-geniculo-occipital (PGO) wave generation (Steriade et al. 1990). Some neurons exhibited low spontaneous firing frequencies (<10 Hz), but most showed high rates of tonic firing in the beta/gamma range (20–80 Hz) in vivo. At present, we do not know if the three types of activity in vivo are related to the different channels modulating gamma band activity in the in vitro PPN. However, it is clear that gamma band oscillations in PPN cells in vitro are mediated by N- and P/Q-type Ca^2+^ channels. A question that arises is, is gamma band activity during waking different from that during REM sleep?

### Waking versus REM sleep

Injections of glutamate into the PPN of the rat were shown to increase both waking and REM sleep, but injections of NMDA increased only waking, while injections of kainic acid (KA) increased only REM sleep (Datta and Siwek 1997; Datta et al. 2001b,b2001a; Datta 2002). Thus, the two states were independently activated by NMDA versus KA receptors. Moreover, the intracellular pathways mediating the two states appear to differ. For example, the CaMKII activation inhibitor, KN-93, microinjected into the PPN of freely moving rats (in vivo) resulted in decreased waking but not REM sleep (Datta et al. 2011). We have shown (in vitro) that beta/gamma band oscillations in PPN neurons are blocked by superfusion of KN-93 (Garcia-Rill et al. 2014), suggesting that at least some cells manifest these oscillations via the CaMKII pathway. Moreover, the stimulant effects of Modafinil, which involve increased electrical coupling, are mediated by the CaMKII pathway since KN-93 inhibits its actions (Garcia-Rill et al. 2007; Urbano et al. 2007). On the other hand, increased ERK1/2 signaling in the PPN is associated with maintenance of sleep via suppression of waking (Desarnaud et al. 2011), while activation of intracellular protein kinase A (PKA) in the PPN instead contributed to REM sleep recovery following REM sleep deprivation (Datta and Desarnaud 2010). These authors showed that during REM sleep, pCREB activation in PPN cholinergic neurons was induced by REM sleep, and that PPN intracellular PKA activation, and a transcriptional cascade involving pCREB, occurred only in cholinergic neurons (Datta et al. 2009). These results suggest that waking in vivo may be modulated by the CaMKII pathway while REM sleep may be modulated by the cAMP/PKA pathway in the PPN (Urbano et al. 2014). Moreover, it appears that the cAMP-dependent pathway phosphorylates N-type channels (Hell et al. 1995), while CaMKII regulates P/Q-type channels (Jenkins et al. 2005). Therefore, the presence of P/Q-type channels is related to CaMKII and waking, while the presence of N-type channels is more related to cAMP and REM sleep (Urbano et al. 2014). The implications from all of these results using a variety of methods is that there is a “waking” pathway mediated by CaMKII and P/Q-type channels, and a “REM sleep” pathway mediated by cAMP/PK and N-type channels.

The evidence described could only have been performed in vitro, since the systematic identification of cells with one versus the other Ca^2+^ channel requires the use of two specific channel blockers tested in the absence of action potentials and fast synaptic transmission. The correlation between cells with P/Q-type channels only manifesting activity during waking but not REM sleep, and those with N-type channels only manifesting activity during REM sleep but not waking, needs to be carried out in vivo. Such experiments will not be simple, but could include the use of intracellular pathway modulators to block or amplify independently the two separate intracellular pathways involved in the action of these Ca^2+^ channels. For example, it may be possible to modulate the CaMKII pathway that supports P/Q-type channel function separately from modulation of the cAMP/PKA pathway that supports N-type channel function. Single cell activity may segregate into that during waking versus REM sleep. Regardless of the complexity, such studies are certainly worthwhile considering the distribution of Ca^2+^ channels described herein. In addition, future studies need to identify the transmitter type of neurons with each type of channel. Our results showed that “N+P/Q” channel-bearing cells were found in all three cell types, “P/Q only” cells also were found in all cell types, as were “N only” cells. Type I cells are typically noncholinergic, 2/3 of type II cells are cholinergic, and 1/3 of type III cells are cholinergic, however, cells with N+P/Q, N only, and P/Q only were represented in all three cell types. This suggests that electrophysiological cell type may be less important than Ca^2+^ channel type in determining beta/gamma band activity during waking versus REM sleep.

## Conclusion

We hypothesize from these results that P/Q-type channels in PPN neurons participate in modulating gamma band activity during waking, while N-type channels modulate gamma band activity during REM sleep. This suggestion will require considerable more research both in vitro and in vivo in order to be confirmed, and is the topic of ongoing studies. The implication of this is that “Wake on” neurons recorded in vivo may have only P/Q-type channels, while “REM on” neurons may have only N-type, while “Wake/REM on” neurons will have both channel types. Such correlations are difficult to investigate, but the results presented here are a first step suggesting that gamma band activity is differentially regulated by two different Ca^2+^ channels that are modulated by two different intracellular pathways. This remains only the first step in a long series of studies required to demonstrate such segregation. The topic is worth pursuing since it may also provide novel therapeutic avenues for differentially treating disorders that are marked by, for example, decreased gamma band activity during waking and increased REM sleep drive, for example, schizophrenia and bipolar disorder (Uhlhaas and Singer 2010; Ozerdem et al. 2011; Urbano et al. 2012; Garcia-Rill et al. 2014).

## Conflict of Interest

None declared.
